# Carga económica de la muerte prematura por suicidio en Colombia entre el 2005 y el 2021

**DOI:** 10.7705/biomedica.7623

**Published:** 2025-05-30

**Authors:** Jean Carlo Pineda-Lozano, Diana Patricia Díaz-Jiménez, Carlos Castañeda-Orjuela

**Affiliations:** 1 Observatorio Nacional de Salud, Instituto Nacional de Salud, Bogotá, D. C., Colombia Instituto Nacional de Salud Instituto Nacional de Salud Bogotá, D. C. Colombia

**Keywords:** suicidio, costos y análisis de costo, carga de enfermedad, costos de enfermedad, salud mental, Colombia, Suicide, costs and cost analysis, burden of disease, cost of illness, mental health, Colombia

## Abstract

**Introducción.:**

El suicidio es un problema de salud pública con consecuencias sociales y económicas significativas. En Colombia, el suicidio afecta principalmente a los jóvenes.

**Objetivo.:**

Estimar los años perdidos de vida potencial y la carga económica asociados con el suicidio en Colombia, entre el 2005 y el 2021.

**Materiales y métodos.:**

Se realizó un estudio retrospectivo para estimar los de años perdidos de vida potencial y la carga económica asociados con el suicidio desde la perspectiva de la sociedad. Se aplicó el método de capital humano modificado y se consideraron dos diferentes escenarios. La información sobre la mortalidad por suicidio y sobre las variables económicas, se recopiló de fuentes oficiales. Se calcularon los costos indirectos por mortalidad prematura, los cuales se expresaron en dólares estadounidenses del 2021 (promedio de la tasa representativa del mercado = COP $ 3.743).

**Resultados.:**

Entre el 2005 y el 2021, se registraron 40.157 muertes por suicidio en Colombia, principalmente en hombres jóvenes de 15 a 29 años. Estas muertes representaron 2’104.731 años perdidos de vida potencial. Los costos totales oscilaron entre USD $ 4.210 millones y USD $ 7.177 millones en las dos circunstancias consideradas, con un costo promedio anual entre USD $ 247,6 y 422,2 millones en cada caso. Los departamentos más poblados tuvieron los mayores costos acumulados por tasas poblacionales. Vaupés, Amazonas y Quindío tuvieron los mayores costos por mil habitantes.

**Conclusiones.:**

Es necesario abordar el suicidio en Colombia desde una perspectiva integral y multidimensional, mediante la inversión en programas de salud mental y enfoques preventivos para reducir la carga económica y el impacto social. Los resultados ofrecen información valiosa para diseñar políticas y estrategias de prevención del suicidio, y resaltan la importancia de enfocarse en grupos poblacionales y regiones vulnerables.

## Abstract (en)

**Introduction.:**

Suicide is a public health problem with significant social and economic consequences. In Colombia, suicide affects mostly young people.

**Objective.:**

To estimate the years of life potentially lost and the economic burden associated with suicide in Colombia between 2005 and 2021.

**Materials and methods.:**

We conducted a retrospective study to estimate the years of life potentially lost and the economic burden related to suicide from the perspective of society. We modified the human capital method and considered two scenarios. Information on suicide mortality and economic variables was collected from official sources, and indirect costs due to premature mortality were calculated. The costs are expressed in 2021 US dollars, considering the average of the 2021 representative market rate (COP $3,743).

**Results.:**

Between 2005 and 2021, 40,157 deaths by suicide were recorded in Colombia, mainly in young men between 15 and 29 years old. These deaths represented 2,104,731 years of life potentially lost. The total economic costs associated with suicide ranged from USD $4.21 billions to USD $7.1 billions in the two scenarios considered, with an average annual cost between USD $247.6 and USD $422.2 millions in each case. The most densely populated departments had the highest accumulated costs by population rates. Vaupés, Amazonas, and Quindío had the highest costs per thousand inhabitants.

**Conclusions.:**

It is necessary to address suicide in Colombia from a comprehensive and multidimensional perspective, investing in mental health programs and preventive approaches to reduce the economic burden and social impact. The results offer valuable information to design suicide prevention policies and strategies and highlight the importance of targeting specific population groups and regions.

El suicidio es un problema grave de salud pública en Colombia y en el mundo, que afecta más a los hombres y genera grandes pérdidas de vida saludable, especialmente en personas en etapa productiva. La Organización Mundial de la Salud (OMS) estimó que en el 2019 ocurrieron 703.000 suicidios en el mundo, con una tasa ajustada por edad de 9,0 por 100.000 habitantes ^1^. En Colombia, para el 2021, se reportaron 2.967 suicidios ^2^.

El estudio del *Global Burden of Disease* (GBD) de la Universidad de Washington, realizado en el 2019, estima que debido al suicidio hubo alrededor de 33,2 millones de años perdidos de vida potencial* en el mundo y 138.000 en Colombia ^3^. Un estudio local reportó 140.383 años perdidos de vida potencial para el 2020, con mayores estimaciones en departamentos como Vaupés y Amazonas, y afectación diferencial en hombres, especialmente entre los 15 y los 29 años ^4^.

Según algunos estudios, tras la pandemia de COVID-19 se ha empeorado la salud mental de la población a nivel global ^5^^,^^6^ y nacional ^7^. Se sabe que la presencia de cualquier trastorno mental, como aquellos relacionados con depresión, ansiedad y rasgos limítrofes de personalidad, se asocian con la ideación suicida ^8^^,^^9^. Un estudio estimó que, en el 2013, el costo del suicidio y el intento de suicidio en Estados Unidos fue de USD $58.400 millones, y la pérdida de productividad representó la mayor parte de ese costo (97,1 %) ^10^.

A pesar de la visibilidad que se le ha dado a la salud mental en los años recientes, existen vacíos en el entendimiento de la problemática desatada por el suicidio y esto puede limitar las acciones que se emprendan en Colombia para prevenir la conducta suicida en la población. En la literatura científica disponible, hay una aproximación a los costos indirectos por mortalidad evitable entre el 1998 y el 2011 en Colombia, en la que se incluyó el suicidio como una de las causas por analizar ^11^. Sin embargo, a la fecha, no se encuentran estudios más recientes en el país que exploren los costos del suicidio en la población, solo existen aproximaciones de las condiciones socioeconómicas como factores influyentes en la conducta suicida ^12^^,^^13^. Otras aproximaciones permiten ver la tendencia del suicidio en rangos de tiempo amplios, con hallazgos importantes sobre el lugar de ocurrencia, el sexo y los métodos utilizados ^14^, pero no profundizan en la estimación económica de la pérdida de vida saludable.

Con el ánimo de visibilizar la problemática desde una perspectiva económica y de salud pública que pueda orientar la toma de decisiones para la promoción y prevención de la salud mental en el país, se desarrolló el presente estudio cuyo objetivo fue estimar los años perdidos de vida potencial y la carga económica según los costos indirectos asociados con la mortalidad por suicidio en Colombia entre el 2005 y el 2021, con desagregación departamental.

## Materiales y métodos

### Diseño del estudio

Se realizó un estudio retrospectivo para estimar los años perdidos de vida potencial y la carga económica -por costos indirectos- desde la perspectiva de la sociedad. Para esto, se utilizó el método de capital humano modificado, que valora el capital incorporado en los trabajadores según el producto interno bruto *per capita* (PIBpc) como una medida estadística de la contribución de cada año de vida a la sociedad ^15^^,^^16^.

Se estimaron los años perdidos de vida potencial por suicidio siguiendo la metodología propuesta por el *Global Burden of Disease Study 2019* (GBD) ^3^. Estos años se estimaron para el total de la población colombiana cuya causa principal de muerte fueron las autolesiones entre los años 2005 y 2021 contrastando la edad en que ocurre la muerte y la esperanza de vida de referencia del GBD. A partir de estos años perdidos de vida potencial, se estimó la pérdida de productividad por mortalidad prematura midiendo la cantidad de años perdidos entre la muerte y los años que hubiera vivido el individuo teniendo en cuenta la edad en la que deja de hacer parte de la fuerza laboral.

### Fuentes de información

La mortalidad por suicidio se obtuvo de las estadísticas vitales del Departamento Administrativo Nacional de Estadística (DANE) ^2^. Se tomaron todas las muertes no fetales que ocurrieron en Colombia en cada uno de los 32 departamentos y su capital, Bogotá -para los fines de este estudio se consideró como otro departamento por el peso relativo de su población-, entre el 1° de enero del 2005 y el 31 de diciembre del 2021, y cuya causa de muerte se catalogó bajo los códigos CIE-10: X60-X64.9, X66-X83.9 y Y87.0. La esperanza de vida utilizada fue la sugerida por el GBD en su tabla de vida de referencia. De igual manera, el DANE fue la fuente de las proyecciones y retroproyecciones poblacionales para el periodo de estudio, ajustadas pospandemia.

En cuanto a las variables económicas, para el 2021, el valor del PIBpc reportado por el Banco de la República de Colombia ^17^ fue de USD $6.241,3 y el valor del salario mínimo anual informado por el Ministerio de Trabajo de Colombia fue de USD $3.660,7 ^18^, contando 13,5 salarios mínimos mensuales equivalentes a los 12 meses laborales más 1,5 salarios mínimos mensuales compuestos por 15 días de vacaciones y dos primas de servicios. Los resultados se presentan en dólares estadounidenses (USD) a precios constantes, utilizando una tasa representativa del mercado promedio de COP $3.743 para el 2021, según los datos del Banco de la República.

### Análisis estadístico

Debido a que en bases de datos de mortalidad se identificaron datos faltantes en las variables de edad y sexo (<1 %), se hizo una imputación de datos con la metodología de *machine learning*. Se utilizaron modelos no paramétricos de clasificación de *random forest* con el paquete *missForest*^19^ del lenguaje de programación R, versión 4.3.0 ^20^. A partir de estas muertes, se estimaron los años perdidos de vida potencial por sexo, grupos de edad quinquenales y departamento de ocurrencia.

Para evitar sobreestimar la pérdida, se hizo una corrección de mitad de periodo, es decir que en cualquier rango de edad se asumió que la muerte ocurrió en promedio a la mitad de dicho intervalo, excepto en aquellos mayores de 80 años, para quienes se asumió el supuesto de que pierden 10 años, ya que es un rango de edad más amplio que el de los otros grupos. El cálculo de los años perdidos de vida potencial con la corrección de mitad de ciclo se explica de esta forma:




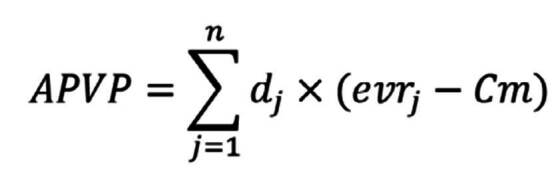




donde:

*d*_*j*_ = defunciones

*evr*_*j*_ = diferencia entre la edad en la que ocurrió la muerte y la expectativa de vida para cada grupo de edad

*Cm* = corrección de mitad de periodo (para evitar sobreestimar la pérdida, se asume que en cualquier rango de edad la muerte ocurrió en la mitad del intervalo).

Los años productivos perdidos de vida potencial (APPVP) se determinaron a partir de la diferencia entre la edad de muerte del individuo y la edad de retiro de la fuerza laboral según la legislación colombiana (57 años para las mujeres y 62 años para los hombres). Finalmente, los costos indirectos por mortalidad prematura (CIMP) se calcularon de la siguiente manera:




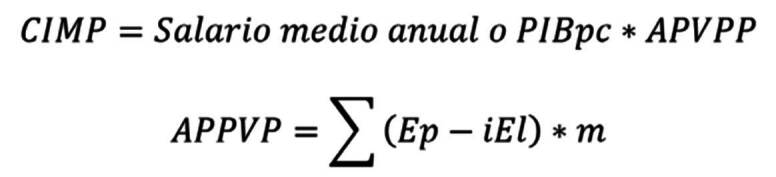




donde:

*Ep* = edad de retiro

*Em* = edad de muerte (ajustada por la mitad del periodo)

*iEl* = la edad de inicio laboral dentro de cada grupo etario dependerá de la edad de muerte del individuo (*Em*) y del inicio de su participación en el mercado laboral, de la siguiente forma:

Si *Em* < 18 (edad mínima de ingreso al mercado laboral), entonces *iEl* = 18

Si *Em* > 18, entonces *iEl* = *iEgm*

*iEgm* = edad de inicio laboral estimada según la del grupo etario al momento de la muerte, ajustada a la mitad del ciclo

*m* = Número de muertes observadas en cada grupo de edad *i*

En la valorización de la carga económica asociada con la mortalidad por suicidio, se estimó el salario o productividad media de dos formas: según el salario mínimo anual y según la productividad media del país (PIBpc en el 2021). En ambos escenarios, se utilizó una tasa de descuento del 3 % para facilitar la comparación de estos resultados con los de otros estudios internacionales ^21^.

## Consideraciones éticas

Según los preceptos de la normativa vigente de Colombia (Resolución 8430 de 1993 del Ministerio de Salud y Protección Social), esta investigación se clasifica como un estudio sin riesgo, teniendo en cuenta que se desarrolló a partir de información secundaria, anonimizada, y que no hubo ninguna intervención.

## Resultados

### Comportamiento del suicidio

Entre el 2005 y el 2021, en Colombia se registraron 40.157 muertes por suicidio (cuadro 1). El 80,7 % de esas muertes correspondieron a hombres, especialmente en los grupos de edad de 15 a 29 años (41,5 %). La mayoría se produjeron en áreas urbanas (77,3 %), concentradas en los departamentos más densamente poblados. El 73,6 % no tenía alguna pertenencia étnica y 38 % de ellos estaban afiliados al régimen subsidiado del sistema de salud.

**Cuadro 1:** Descripción de las variables sociodemográficas de las personas que murieron por suicidio en Colombia entre el 2005 y el 2021 (N = 40.157)

Característica		n (%)	
Sexo			
	Mujeres	7.766	(19,3)
	Hombres	32.391	(80,7)
Edad			
	0-14	1.578	(3,9)
	19-49	28.801	(71,7)
	50-64	5.857	(14,6)
	≥ 65	3.921	(9,8)
Lugar de residencia			
	Urbano	31.049	(77,3)
	Rural	7.935	(19,8)
	Sin información	1.173	(2,9)
Pertenencia étnica			
	Ninguno	29.551	(73,6)
	Negro, mulato, afro	992	(2,5)
	Indígena	916	(2,3)
	Otros	32	(0,1)
	Sin información		
Régimen de salud		8.666	(21,6)
	Subsidiado	15.247	(38,0)
	Contributivo	10.042	(25,0)
	Especial	700	(1,7)
	Sin información	14.168	(35,3)

### Años perdidos de vida potencial por suicidio

**Figura 1:**
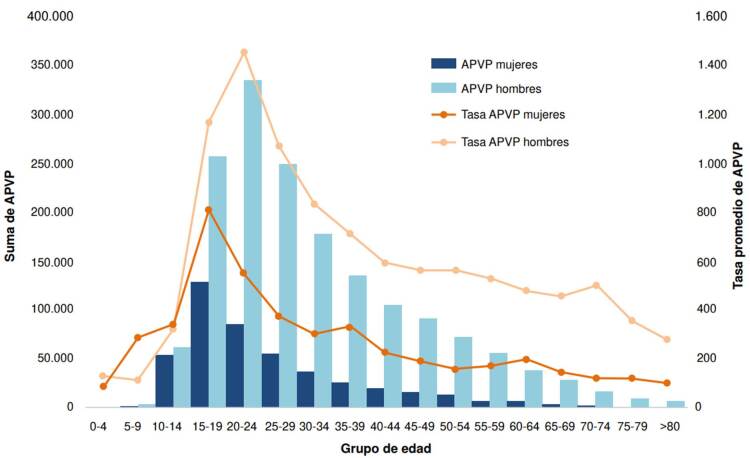
Total de años perdidos de vida potencial (APVP) entre el 2005 y el 2021, y tasa promedio por grupo de edad y sexo en Colombia

Las muertes por suicidio representaron 2’104.731 de años perdidos de vida potencial (figura 1). El total y la tasa promedio anual de estos años perdidos en el periodo analizado, fueron mayores en hombres y en los grupos de edad de 15 a 29 años.

**Figura 2:**
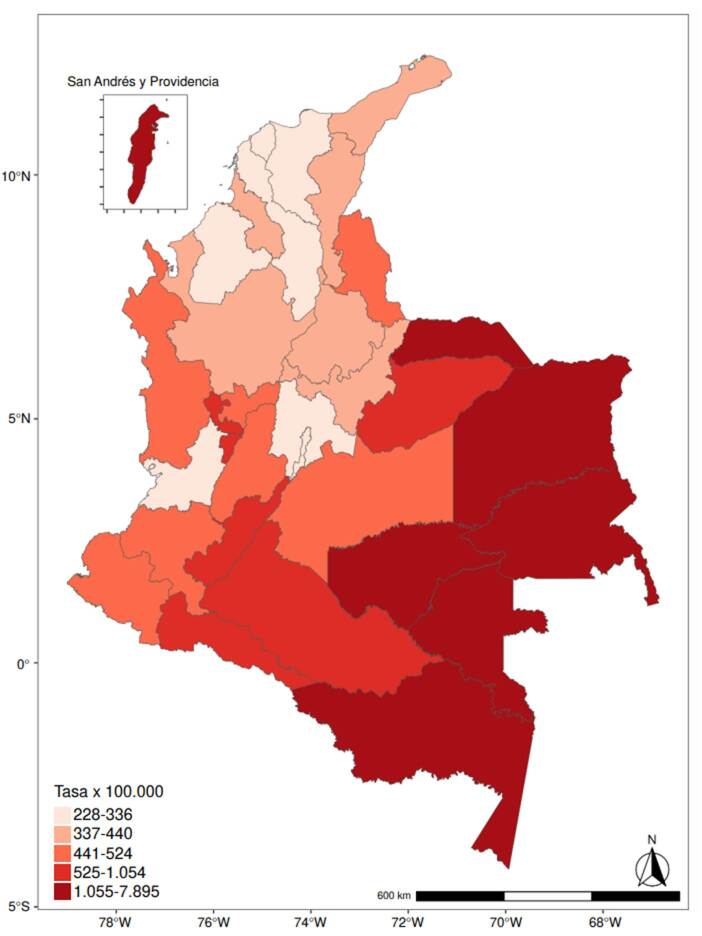
Tasa promedio anual de años perdidos de vida potencial por departamento en Colombia entre el 2005 y el 2021

La tasa promedio anual de años perdidos de vida potencial por 100.000 habitantes para el país fue de 587,7. Los departamentos con las tasas promedio más altas fueron Vaupés (7.895), Guainía (4.719) y Amazonas (3.198), mientras que las tasas más bajas se presentaron en Bogotá (228,9), Bolívar (249,9) y Atlántico (250,2) (figura 2).

### Carga económica del suicidio

**Figura 3:**
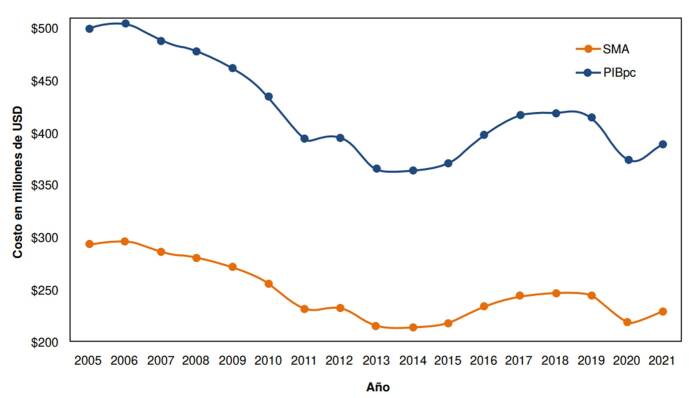
Costos indirectos por mortalidad prematura a causa de suicidio en Colombia entre el 2005 y el 2021, en dos circunstancias, representados en millones de dólares (USD), ajustados a la tasa representativa del mercado del 2021

Entre el 2005 y el 2021, las muertes reportadas representaron un costo total de USD $4.210 millones en el caso del salario mínimo anual y de USD $7.177 millones en el caso del PIBpc, con un costo promedio anual de COP $247,6 millones (DE = 27,4) y USD $422,2 millones (DE = 46,7), respectivamente. La tendencia de los costos fue decreciente hasta el 2013; a partir del 2015, se observó un incremento que va hasta el 2019, antes de la pandemia de la COVID-19 (figura 3).

**Figura 4:**
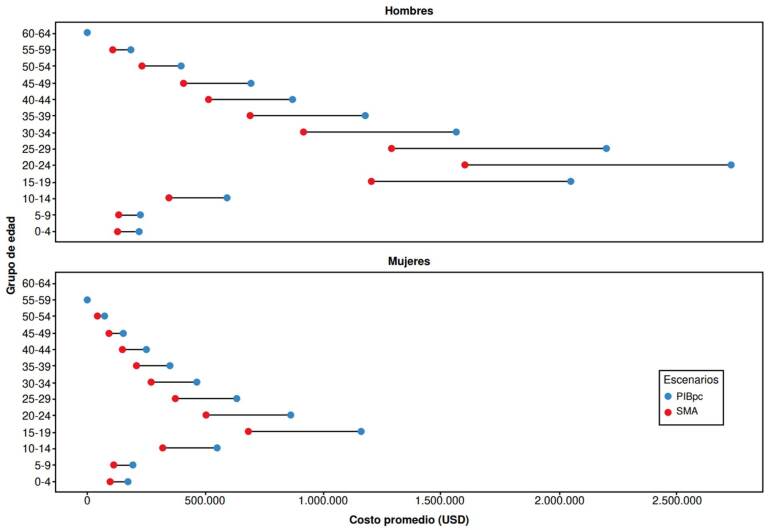
Costos indirectos promedio por mortalidad prematura debido al suicidio según grupo de edad y sexo en Colombia entre el 2005 y el 2021 (en dólares ajustados a la tasa representativa del mercado del 2021)

Las muertes de hombres entre los 20 y los 24 años representaron los costos más altos en ambos escenarios, con un costo promedio de USD $1’587.149 en el salario mínimo anual y de $2’705.981 en el PIBpc (figura 4), y un costo acumulado de USD $793,5 (salario mínimo anual) y de USD $1.352,9 millones (PIBpc) (anexo 1), entre el 2005 y el 2021.En el caso de las mujeres, en el grupo de los 15 a los 19 años se concentró el mayor costo promedio, de USD $667.250 (salario mínimo anual) y de USD $1.137.615 (PIBpc) (figura 4), y un costo acumulado de USD $324,1 (salario mínimo anual) y de USD $484,6 millones (PIBpc) en el periodo analizado (anexo 1). Los costos fueron USD $0 en el grupo de 60 a 64 años en ambos sexos, dada la edad de pensión en el país.

A nivel departamental, el costo acumulado del periodo y el promedio anual para ambos casos, se concentraron en los entes territoriales con mayor densidad poblacional, tales como Antioquia (16,3 %), Bogotá (13,1 %) y Valle del Cauca (9,1 %). Al ajustar los costos por población en tasas por 1.000 habitantes, los departamentos de Vaupés, Amazonas y Quindío tuvieron el costo más alto en los dos casos (cuadro 2).

**Cuadro 2:** Costo total del periodo y costo promedio anual de suicidios por departamento de ocurrencia en Colombia entre el 2005 y el 2021 (en dólares ajustados a la tasa representativa del mercado del 2021)

Ente territorial	Costo total periodo, SMA^a^	Costo total periodo, PIBpc^a^	Costo promedio anual, SMA^a^	Costo promedio anual, PIBpc^a^	Costo total periodo, SMA por 1. 000 habitantes^b^	Costo total periodo, PIBpc por 1.000 habitantes^b^
Vaupés	17,84	30,41	1,19	2,03	13,28	22,63
Amazonas	16,79	28,63	0,99	1,68	6,82	11,63
Quindío	79,69	135,86	4,69	7,99	4,69	7,99
Nariño	239,36	408,10	14,08	24,01	4,65	7,93
Huila	152,38	259,79	8,96	15,28	4,34	7,41
Arauca	37,45	63,85	2,20	3,76	4,29	7,32
Tolima	170,56	290,80	10,03	17,11	4,05	6,91
Caldas	124,97	213,06	7,35	12,53	3,93	6,70
Risaralda	111,31	189,78	6,55	11,16	3,81	6,49
Cauca	177,26	302,22	10,43	17,78	3,80	6,48
Putumayo	41,31	70,43	2,43	4,14	3,72	6,34
Caquetá	43,65	74,42	2,57	4,38	3,40	5,79
Antioquia	684,19	1.166,50	40,25	68,62	3,39	5,79
Boyacá	123,79	211,05	7,28	12,41	3,16	5,38
Norte de Santander	142,35	242,69	8,37	14,28	2,95	5,02
Meta	94,30	160,77	5,55	9,46	2,86	4,88
Guaviare	8,00	13,64	0,47	0,80	2,77	4,73
Valle del Cauca	384,73	655,93	22,63	38,58	2,76	4,70
Santander	186,18	317,43	10,95	18,67	2,69	4,58
Casanare	36,05	61,47	2,12	3,62	2,66	4,54
Bogotá, D. C.	550,30	938,22	32,37	55,19	2,36	4,02
Guainía	3,56	6,06	0,25	0,43	2,24	3,82
Cundinamarca	207,69	354,09	12,22	20,83	2,13	3,63
Cesar	82,36	140,42	4,84	8,26	2,10	3,58
Vichada	6,78	11,56	0,45	0,77	1,93	3,30
Sucre	49,61	84,59	2,92	4,98	1,70	2,89
Magdalena	69,37	118,28	4,08	6,96	1,61	2,74
Córdoba	85,92	146,48	5,05	8,62	1,52	2,60
Atlántico	122,81	209,38	7,22	12,32	1,52	2,60
Bolívar	98,41	167,78	5,79	9,87	1,49	2,55
San Andrés	2,59	4,42	0,20	0,34	1,36	2,31
La Guajira	39,28	66,96	2,31	3,94	1,35	2,30
Chocó	18,68	31,84	1,10	1,87	1,08	1,84
Total	4 209,51	7.176,93	247,62	422,17	2,74	4,67

SMA: salario mínimo anual

^a^ Millones de USD

^b^ Miles de USD

Aunque los departamentos de Atlántico y Bolívar tienen costos por 1.000 habitantes relativamente bajos, en comparación con los otros departamentos, su costo total supera los USD $98 millones y el costo promedio anual está por encima de los USD $5,8 (salario mínimo anual) y USD $9,8 millones (PIBpc).

## Discusión

El presente estudio se centró en la estimación de años perdidos de vida potencial y los costos asociados con el suicidio en Colombia del 2005 al 2021. Los resultados revelaron el comportamiento del suicidio, la carga económica y la distribución geográfica de los costos. Estos hallazgos proporcionan información valiosa para comprender el impacto económico de este fenómeno en el país y pueden ser utilizados como insumos para el diseño de estrategias de prevención y políticas públicas. Se observó que, entre el 2005 y el 2021, se registraron 40.157 muertes por esta causa en Colombia, con predominio en hombres entre los 15 y los 29 años, lo cual coincide con otras publicaciones ^1^^,^^22^^,^^23^. Se presentaron más casos en el régimen subsidiado de afiliación al sistema de salud, lo que podría interpretarse como un indicador proxi de condiciones socioeconómicas desfavorables; esto refuerza la relación entre la pobreza, el acceso limitado a servicios de salud mental y el riesgo de suicidio. Este hallazgo subraya la necesidad de mejorar el acceso a los servicios de salud mental en poblaciones vulnerables, especialmente entre quienes están afiliados al régimen subsidiado, como parte de una estrategia integral de prevención.

El análisis de la carga económica del suicidio reveló que las muertes reportadas representaron un costo total mínimo de USD $4.210 millones y máximo de USD $7.177 millones. Al desglosar los costos por sexo y grupo de edad, se encontró que los hombres de 20 a 24 años generaron los costos más altos en los dos casos contemplados (salario mínimo anual y PIBpc). Por otro lado, en el caso de las mujeres, el grupo de 15 a 19 años fue el que concentró el mayor costo promedio. Estos resultados resaltan la importancia de abordar la prevención del suicidio en grupos de edad específicos, especialmente en adolescentes y adultos jóvenes.

La distribución geográfica de los costos por suicidio mostró que los sitios con mayor densidad poblacional, como Antioquia, Bogotá y Valle del Cauca, presentaron los mayores costos acumulados y promedio anuales. Sin embargo, al generar las tasas de costos por mil habitantes, se encontró que los departamentos de Vaupés, Amazonas y Quindío tuvieron los costos más altos. Otros autores encontraron que el riesgo de morir por suicidio entre el 2010 y el 2013 fue mayor en Vaupés, Huila y Quindío ^24^. Estos hallazgos se relacionan con los resultados del presente estudio, excepto en el caso del departamento del Amazonas, donde, al parecer, en los años más recientes han aumentado las cifras de suicidio. En la literatura científica se ha evidenciado que habitar en áreas rurales aumenta el riesgo de morir por suicidio (HR = 2,56; IC _95%_: 2,04-3,20) cuando ya se han tenido intentos previos de suicidio ^25^. Estos resultados sugieren la necesidad de implementar medidas preventivas y de atención en áreas con mayor carga económica relativa, incluso si su densidad poblacional es menor.

En otro estudio en Colombia, se reportaron costos indirectos por mortalidad prematura debido a muertes evitables entre 1998 y el 2011. Los hallazgos demostraron que el suicidio fue la cuarta causa de muerte que más costos indirectos representó para el país (4 % del total), con un rango de USD $2.800 a USD $5.300 millones ^11^. Estos valores son inferiores a los reportados en el presente estudio, pero tal vez se deba al periodo contemplado: los autores incluyen datos de mortalidad de 14 años (19982011), mientras que nosotros evaluamos datos de mortalidad de 17 años (2005-2021). En los Estados Unidos se reportaron USD $53.047 millones por costos indirectos relacionados con mortalidad por suicidio en el 2013, antes de ajustar por subregistro ^10^, es decir, USD $1,28 millones por suicidio ocurrido, mientras que en el presente análisis, para el mismo año, el costo por suicidio (en dólares internacionales, ajustados por paridad de poder adquisitivo en el 2013) ^26^ fue mínimo de USD $0,36 y máximo de USD $0,61 millones. Estas discrepancias se explican por los diferentes enfoques metodológicos utilizados, y porque el PIBpc de Estados Unidos es mucho más alto en comparación con el de Colombia y fue el que se usó de base para estimar los costos indirectos.

La carga económica del suicidio en Colombia es un fenómeno complejo y multifactorial. En este estudio se destaca la necesidad de abordar esta problemática desde una perspectiva multidimensional, que tome en cuenta factores sociales, económicos y culturales. Es fundamental considerar las particularidades de cada grupo poblacional y región para desarrollar estrategias efectivas y ajustadas a sus necesidades específicas. La inversión en salud mental es crucial para abordar el problema del suicidio en Colombia y se deben destinar recursos adecuados para la promoción de la salud mental, la prevención del suicidio y la atención de las personas en riesgo. Estos esfuerzos incluyen la mejora del acceso a los servicios de salud mental, la capacitación de profesionales de la salud y la sensibilización pública sobre la importancia de la salud mental. Si bien la atención y el tratamiento son importantes, se debe hacer mayor énfasis en la prevención del suicidio. Los programas preventivos pueden incluir la detección temprana de factores de riesgo, la promoción de habilidades de afrontamiento saludables, la reducción del estigma asociado a los problemas de salud mental y la implementación de políticas de bienestar que aborden las causas subyacentes del suicidio ^27^^,^^28^.

Estos hallazgos son relevantes para las Américas y pueden servir como referencia para otros países, ya que esta es la única región donde las tasas de suicidio aumentaron durante 20 años (2000-2019), en contraste con el descenso observado en el resto del mundo según los datos proporcionados por la OMS ^1^. La misma fuente destaca que el suicidio constituye un problema de salud pública que afecta a la mayoría de los países de la región. Hasta el 2019, el 77 % de las muertes por suicidio tuvieron lugar en naciones con bajos y medianos ingresos.

Este análisis tiene algunas limitaciones, entre las que se incluye su enfoque en los costos indirectos del suicidio que, si bien incluyen aspectos como la pérdida de productividad laboral y el impacto en la economía en general, no contemplan otros costos directos e indirectos, como los gastos médicos y los efectos psicosociales en las familias y las comunidades afectadas. Por lo tanto, los resultados presentados en este artículo representan una estimación parcial de la carga económica total del suicidio en Colombia.

Asimismo, el estudio depende de los datos de mortalidad y de variables económicas reportados por fuentes oficiales, lo que podría llevar a subestimar o sobreestimar los resultados debido a posibles errores o falta de información precisa. Además, la metodología utilizada para calcular los años perdidos de vida potencial podría no capturar todas las complejidades de la pérdida de vida saludable y omitir factores como la calidad de vida durante esos años, por ejemplo, en los casos de ideación o intento de suicidio no consumado. Otra limitación del estudio es la falta de análisis longitudinal, pues este se basa en un enfoque transversal y no se consideran posibles cambios en los patrones de suicidio y carga económica a lo largo del tiempo.

Los costos estimados en este estudio se presentan en dólares estadounidenses del 2021, ajustados según la tasa representativa del mercado de ese año. Este enfoque asegura la coherencia en la comparación temporal y evita la distorsión causada por la inflación. Por otro lado, aunque asumir tasas de empleo del 100 % en los diferentes grupos económicamente activos podría sobreestimar los costos, realmente corresponde a una valoración más justa de la productividad perdida. Esto se debe a la implementación de un enfoque de capital humano a diferencia de uno más utilitarista -como el de costos de fricción- que subestima las perdidas, ajustando por tasas reales de desempleo o subempleo, las cuales son fallas del mercado laboral.

Finalmente, no se exploraron factores contextuales, culturales, psicológicos o sociales que podrían influir en las tasas de suicidio y en la carga económica, lo que limita la comprensión completa de los resultados.

Este estudio proporciona una visión detallada de los costos asociados con el suicidio en Colombia en términos de la pérdida de vida productiva, y destaca la importancia de abordar este problema desde una perspectiva integral que considere tanto los aspectos socioeconómicos como los factores de riesgo individuales y comunitarios. Los resultados obtenidos pueden utilizarse para informar y respaldar la implementación de políticas de prevención del suicidio, generar estrategias de prevención, promover una mayor conciencia sobre este problema de salud pública y resaltar la importancia de enfocarse en grupos poblacionales específicos y las regiones con mayor carga económica. Sin embargo, es necesario realizar investigaciones adicionales que aborden los costos directos e indirectos no contemplados en este estudio, para obtener una imagen más completa del costo económico y social del suicidio en el país.

## Figures and Tables

**Anexo 1:**
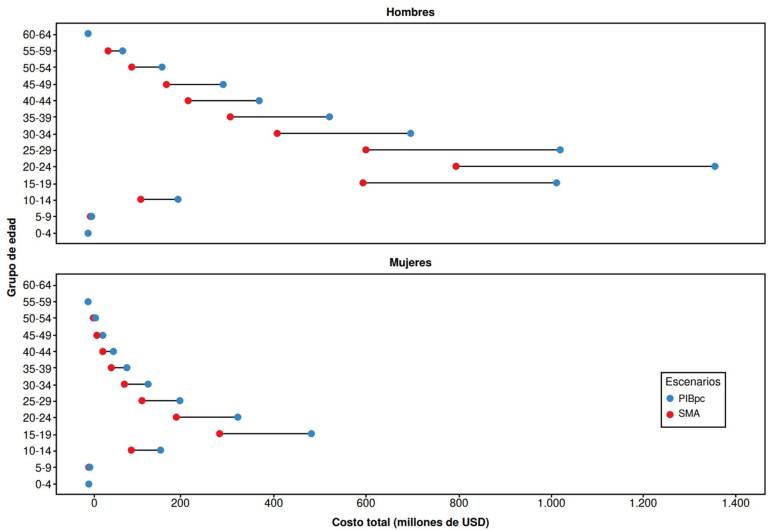
Costos indirectos totales por mortalidad prematura debido al suicidio, según grupo de edad y sexo en Colombia, entre el 2005 y el 2021 (en dólares ajustados a la tasa representativa colombiana del 2021).

## Footnotes

**Citación:** Pineda-Lozano JC, Díaz-Jiménez DP, Castañeda- Orjuela C. Carga económica de la muerte prematura por suicidio en Colombia entre el 2005 y el 2021. Biomédica. 2025;45:215-27. https://doi.org/10.7705/biomedica.7623

## References

[1] World Health Organization (2021). Suicide Worldwide in 2019 Global Health Estimates.

[2] Departamento Administrativo Nacional de Estadística - DANE (2023). Estadísticas vitales.

[3] Abbafati C, Abbas KM, Abbasi-Kangevari M, Abd-Allah F, Abdelalim A, Abdollahi M (2020). Global burden of 369 diseases and injuries in 204 countries and territories, 1990-2019: A systematic analysis for the Global Burden of Disease Study 2019. Lancet.

[4] Instituto Nacional de Salud (2022). Panorama de eventos en salud pública.

[5] Buitrago Ramírez F, Ciurana Misol R, Fernández Alonso M del C, Tizón García JL. (2020). Salud mental en epidemias: una perspectiva desde la atención primaria de salud española. Aten Primaria.

[6] Brooks SK, Webster RK, Smith LE, Woodland L, Wessely S, Greenberg N (2020). The psychological impact of quarantine and how to reduce it: Rapid review of the evidence. Lancet.

[7] Observatorio Nacional de Salud (2021). COVID-19: progreso de la pandemia y sus desigualdades en Colombia.

[8] Martínez Silva PA, Dallos Arenales MI, Prada AM, Rodríguez van der Hammen MC, Mendoza Galvis N. (2020). An explanatory model of suicidal behaviour in indigenous peoples of the department of Vaupés, Colombia. Rev Colomb Psiquiatr.

[9] Ministerio de Salud y Protección Social Subdirección de Enfermedades no Transmisibles (2018). Boletín de salud mental: Conducta suicida.

[10] Shepard DS, Gurewich D, Lwin AK, Reed GA, Silverman MM (2016). Suicide and suicidal attempts in the United States: Costs and policy implications. Suicide Life Threat Behav.

[11] Díaz-Jiménez D, Castañeda-Orjuela C, Castillo-Rodríguez L, De La Hoz-Restrepo F. (2015). Economic costs analysis of the avoidable mortality in Colombia 1998-2011. Value Health Reg Issues.

[12] Castro-Díaz S, Gómez-Restrepo C, Gil F, Uribe Restrepo M, Miranda C, de la Espriella M (2013). Factores de riesgo para ideación suicida en pacientes con trastorno depresivo en Colombia. Rev Colomb Psiquiatr.

[13] Buendía JA, Restrepo Chavarriaga GJ, Zuluaga AF. (2020). Social and economic variables related to Paraquat self-poisoning: An ecological study. BMC Public Health.

[14] Chaparro-Narváez P, Díaz-Jiménez D, Castañeda-Orjuela C. (2019). Tendencia de la mortalidad por suicidio en las áreas urbanas y rurales de Colombia, 1979-2014. Biomedica.

[15] Drummond M. (1992). Cost-of-illness studies: A major headache?. PharmacoEconomics.

[16] Chen S, Zhao J, Lee SB, Kim SW. (2022). Estimation of relative risk of mortality and economic burden attributable to high temperature in Wuhan, China. Front Public Health.

[17] Banco de la República Producto Interno Bruto (PIB), 2021.

[18] Ministerio de Trabajo Trabajadores colombianos tendrán salario mínimo de $908.526 más auxilio de transporte de $106.454 en el 2021.

[19] Stekhoven DJ, Bühlmann P. (2012). MissForest-non-parametric missing value imputation for mixed- type data. Bioinformatics.

[20] RStudio Team (2020). RStudio: Integrated Development for R. RStudio.

[21] Khorasani E, Davari M, Kebriaeezadeh A, Fatemi F, Akbari Sari A, Varahrami V. (2022). A comprehensive review of official discount rates in guidelines of health economic evaluations over time: The trends and roots. Eur J Health Econ.

[22] Chang Q, Yip PSF, Chen YY. (2019). Gender inequality and suicide gender ratios in the world. J Affect Disord.

[23] Cardona Arango D, Medina-Pérez ÓA, Cardona Duque DV (2016). Caracterización del suicidio en Colombia, 2000-2010. Rev Colomb Psiquiatr.

[24] Rodríguez-Hernández JM, Rocha-Buelvas A, Mendieta-Izquierdo G, Hidalgo-Troya A. (2018). Riesgo de muerte por suicidio en población colombiana 2000-2013. Ciênc Saúde Coletiva.

[25] Castro Moreno LS, Fuertes Valencia LF, Pacheco García OE, Muñoz Lozada CM. (2021). Factores de riesgo relacionados con intento de suicidio como predictores de suicidio, Colombia 2016-2017. Rev Colomb Psiquiatr.

[26] World Bank PPP conversion factor, GDP (LCU per international $) - Colombia, United States. 2023. PPP conversion factor, GDP (LCU per international $) - Colombia, United States.

[27] Platt S, Niederkrotenthaler T. (2020). Suicide prevention programs: Evidence base and best practice. Crisis.

[28] Fughal F, Gorton HC, Michail M, Robinson J, Saini P. (2021). Suicide prevention in primary care: The opportunity for intervention. Crisis.

